# Association of serum gastric inhibitory polypeptide and pancreatic polypeptide levels with prolonged esophageal acid exposure time in refractory gastroesophageal reflux disease

**DOI:** 10.1097/MD.0000000000015965

**Published:** 2019-06-07

**Authors:** Jing Chen, Baona Guo, Zihao Guo, Li Li, Jiali Jiang, Yutao Zhan, Jixiang Wu, Chuan Zhang

**Affiliations:** a Department of Gastroenterology; b Department of General Surgery, Beijing Tongren Hospital, Capital Medical University, Beijing, China.

**Keywords:** acid exposure time, gastric inhibitory polypeptide, gastroesophageal reflux disease, pancreatic polypeptide

## Abstract

**Background:**

Acid exposure time (AET) prolongation plays an important role in the pathogenesis of gastroesophageal reflux disease (GERD). Gastric inhibitory polypeptide (GIP) and pancreatic polypeptide (PP) participate in the regulation of gastric acid secretion, blood glucose and lipid levels, and food intake. In this study, we evaluated the serum GIP and PP levels in refractory GERD patients and analyzed their metabolic and motility characteristics.

**Methods:**

Seventy-three refractory GERD patients were enrolled in this study from September 2015 to September 2017. We investigated the clinical characteristics, severity, and duration of GERD symptoms. High-resolution manometry and 24 hours impedance-pH monitoring were performed to assess esophageal motility and reflux parameters. The patients were divided into the AET− group (AET <4.2%) and AET+ group (AET >4.2%). GIP and PP levels were determined in all subjects and their associations with other parameters evaluated.

**Results:**

Age and GERDQ score were significantly higher (*P* < .05) and acid reflux and heartburn more frequent in the AET+ group than in the AET− group. The contraction front velocity was increased in the AET− group, while there was no significant difference in the distal contraction integral, peristalsis interruption, distal latency, or resting pressures of the lower and upper esophageal sphincters between the 2 groups (*P* > .05). The serum levels of GIP (*P* = .003) and PP (*P* = .012) were significantly increased in the AET+ group. Increased GIP and PP levels were associated with abnormal upright AET (correlation coefficients 0.307 and 0.233, *P* = .008 and *P* = .047). There was a positive correlation between GIP and triglyceride levels (correlation coefficient 0.279, *P* = .017).

**Conclusion:**

The serum levels of GIP and PP in refractory GERD patients with prolongation of AET are significantly elevated, mainly in the upright position.

## 1 Introduction

The prevalence of gastroesophageal reflux disease (GERD) has increased recently, particularly in North America and East Asia since 1995.^[1]^ Proton-pump inhibitors (PPIs) are important for the treatment of GERD as they reduce the acidity of refluxate. However, these agents are unsatisfactory in a subset of patients, who are considered to be unresponsive to PPIs. The prevalence of this emerging phenomenon, defined as refractoriness to PPIs, is 10% to 40%.^[2,3]^

The pathogenesis of GERD is multi-factorial and not fully understood. Alterations of the gut–brain interaction lead to the development of various gastrointestinal (GI) disorders, including GERD.^[4]^ Several peptide hormones play an important role in regulating food intake, gastric acid secretion, GI motility, and energy balance. Whether these humoral factors contribute to the development and progression of GERD is unclear.

In this study, we compared the circulating levels of 2 peptide hormones, gastric inhibitory polypeptide (GIP) and pancreatic polypeptide (PP), in refractory GERD patients. We also explored the relationships between the serum GIP and PP levels and objective indicators of GERD (ie, high-resolution manometry [HRM] of the esophagus and ambulatory 24 hours impedance-pH [MII-pH] monitoring), as well as the symptom profile, medical history, and carbohydrate and fat metabolism of GERD patients.

## 2 Materials and methods

### 2.1 Patients

This study included 114 patients with refractory GERD symptoms that persisted after 8 weeks of standard PPI therapy (single daily dose) during 2015 to 2017. Seventy-three patients underwent upper GI endoscopy to identify reflux esophagitis (RE) and other organic abnormalities. Patients with previous gastroesophageal surgery or hiatal hernia were excluded. The patients underwent HRM and ambulatory 24 hours MII-pH monitoring after discontinuation of PPI for 7 days. The study was performed in accordance with the ethical standards specified by the Declaration of Helsinki and its amendments and approved by the Ethics Committee of Beijing Tongren Hospital, Capital Medical University. All participants provided written informed consent before enrollment in the study.

### 2.2 Data collection

We obtained information using questionnaires on age, sex, body mass index (BMI, kg/m^2^), waist circumference at the level of the navel during minimal respiration, diabetes history, cigarette smoking status, and alcohol consumption. Blood triglyceride (TG), total cholesterol (TC), high-density lipoprotein cholesterol (HDL-C), low-density lipoprotein cholesterol (LDL-C), and fasting blood glucose levels were measured. RE was graded from A to D according to the Los Angeles (LA) classification system using standard comparator photos. Upper GI endoscopy was performed by experienced endoscopists who were blinded to the questionnaire results. Patients defined their symptoms using the validated GerdQ score on a 4-point (0–3) Likert scale (0 = never, 1 = 1 day, 2 = 2–3 days, and 3 = 4–7 days during the previous week) rating the frequencies of 4 positive predictors of GERD (heartburn, regurgitation, sleep disturbance due to nocturnal reflux symptoms, and use of over-the-counter medications to control reflux symptoms) and a reverse Likert scale (3–0) rating 2 negative predictors of GERD (epigastric pain and nausea), giving a total GerdQ score range of 0 to 18. A GerdQ score ≥9 was considered to indicate positivity.^[5]^

### 2.3 HRM and ambulatory 24 hours MII-pH monitoring

HRM with esophageal pressure topography studies was performed using a Solar GI HRM (medical measurements systems [MMS], Enschede, The Netherlands). The lower esophageal sphincter (LES) pressure, distal contractile integral (DCI), contractile front velocity (CFV), distal latency (DL), 4 seconds integrated relaxation pressure (IRP4s), upper esophageal sphincter (UES) pressure, and the presence of motility disorders in each subject was assessed by 10 saline (5 mL) swallows. The diagnosis of esophageal dysmotility was made according to the criteria of the Chicago classification (CC) ver. 3.0.^[6]^

Ambulatory 24 hours MII-pH monitoring was performed using the multi-channel intraluminal impedance ambulatory system (MMS). The system includes a portable data logger with MII-pH amplifiers. After calibration in pH 4.0 and 7.0 buffer solutions, the MII-pH catheter was positioned in the esophageal body, with the pH electrode at 5 cm and the impedance channels at 3, 5, 7, 9, 15, and 17 cm proximal to the LES. Subjects were required to fast overnight and maintain normal activities during the 24 hours before monitoring. Symptoms, meals, and postures were recorded by pressing the event buttons.

The MII-pH monitoring data were analyzed by 2 investigators (Z Guo and B Guo). Reflux episodes were characterized as acidic, weakly acidic, or nonacidic and liquid, mixed, or gas according to a consensus report on detection and definitions. An abnormal acid exposure time (AET) was defined as that >4.2%.^[7]^ The patients were classified into 2 groups based on their ambulatory MII-pH monitoring results:

(1) those with an AET <4.2% were included in the normal esophageal AET group (AET− group)
(2) and those with an AET ≥ 4.2% in the abnormal esophageal AET group (AET+ group).

The DeMeester score includes and weighs 6 different parameters: the total percentage of time during which the pH <4, the percentage of time with pH <4 in the upright position, the percentage of time with pH <4 in the supine position, the total number of reflux episodes, the total number of reflux episodes lasting longer than 5 minutes, and the duration of the longest reflux episode. A symptom index (SI) was used to determine symptom–reflux associations; an SI ≥50% was considered positive.^[8]^

Data acquisition, online visualization, and signal processing were performed using a commercially available manometric system (MMS database software version 9.3).

### 2.4 Serum measures

Serum samples were collected at the time of examination and stored at −80°C. GIP and PP levels were quantified in duplicate using the Milliplex Human Metabolic Hormone Magnetic Bead Panel (Merck Millipore, Billerica, MA) and a Luminex instrument (Eve Technologies, Calgary, Canada).

### 2.5 Statistical analysis

Normally distributed continuous data are expressed as means ± standard deviation and were compared by Student *t* test. For skewed variables, data are expressed as medians with the interquartile range and compared using the Wilcoxon 2-sample test. Categorical data are expressed as percentages and were analyzed by Pearson *χ*^2^ test or Fisher exact test, as appropriate. Univariate relationships between peptide hormone levels and risk factors were assessed by Spearman correlation. Receiver operating characteristic (ROC) curves with associated 95% confidence intervals (CI) were used to determine the optimal cut-off values for diagnosis of abnormal AET and their associated sensitivities and specificities. A value of *P* < .05 was considered to indicate statistical significance. All statistical analyses were performed using SPSS ver. 22 (SPSS Inc, Chicago, IL).

## 3 Results

### 3.1 Study population

**Figure 1:**
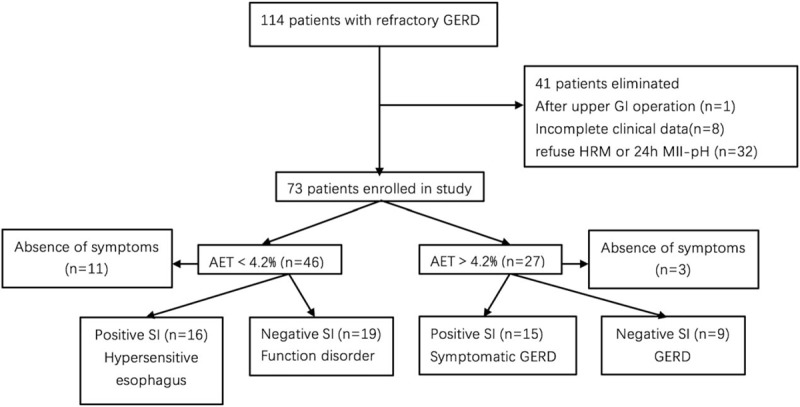
Patient recruitment flowchart.

A total of 114 patients with refractory GERD symptoms were recruited initially (Fig. 1), of whom 73 consecutive subjects (61.37 ± 11.34 years, 65.75% women) had complete HRM and 24 hours MII-pH monitoring data and were included in the analysis (Table 1). The symptom duration of these patients was 0.5 to 60 years. Their chief complaints were heartburn and acid regurgitation. Other complaints included abdominal pain (23.29%), abdominal distention (23.29%), chest back pain (26.03%), cough (17.81%), pharyngodynia (26.03%), and dysphagia (6.85%). Fifty-nine patients had erosive esophagitis of LA grades A (39/59), B (14/59), C (6/59), and D (0/59). Fourteen patients had chronic gastritis without erosive esophagitis.

**Table 1:**
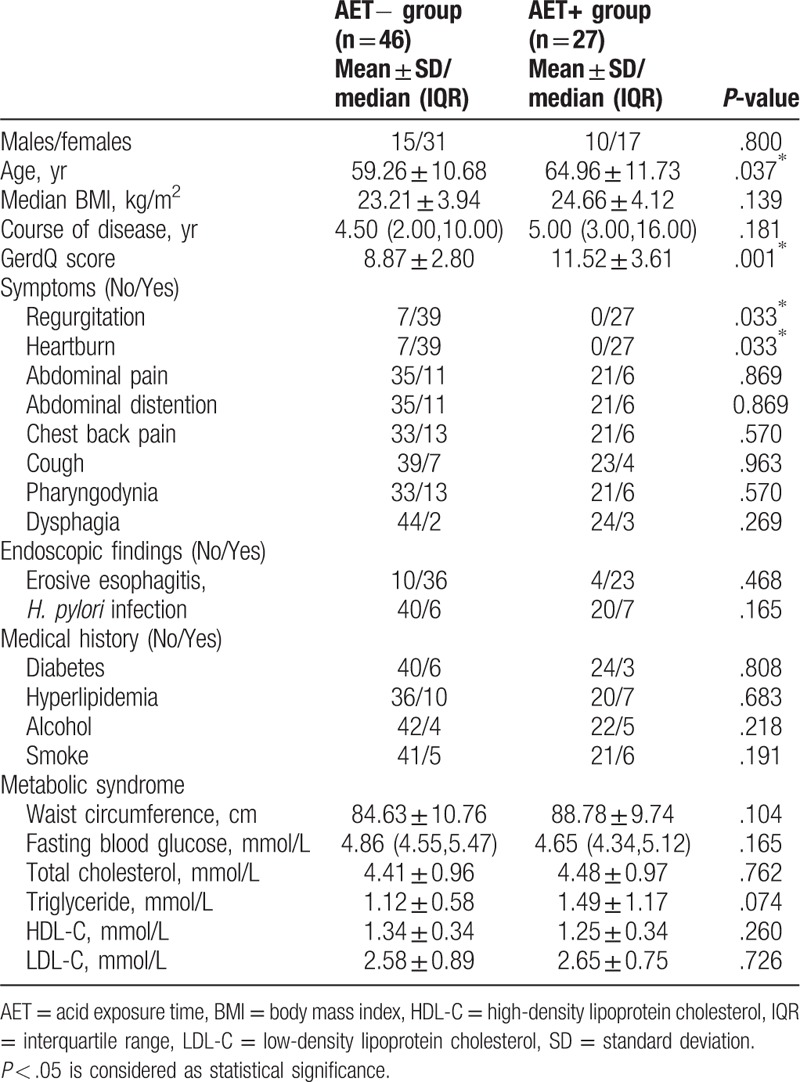
Demographic, endoscopic findings, medical history, and metabolic syndrome of patients with refractory GERD.

The 2 groups differed significantly in terms of age (59.26 ± 10.68 and 64.96 ± 11.73 years, *P* = .037) and GerdQ score (8.87 ± 2.80 and 11.52 ± 3.61, *P* = .001) respectively between AET− and AET+ group. Regurgitation and heartburn were more prevalent in the AET+ group, while there were no significant differences in other symptoms, endoscopic findings, or medical histories (all *P* > .05). Waist circumference and fasting blood glucose, TG, TC, HDL-C, and LDL-C levels were not associated with AET.

### 3.2 Serum levels of GIP and PP

**Figure 2:**
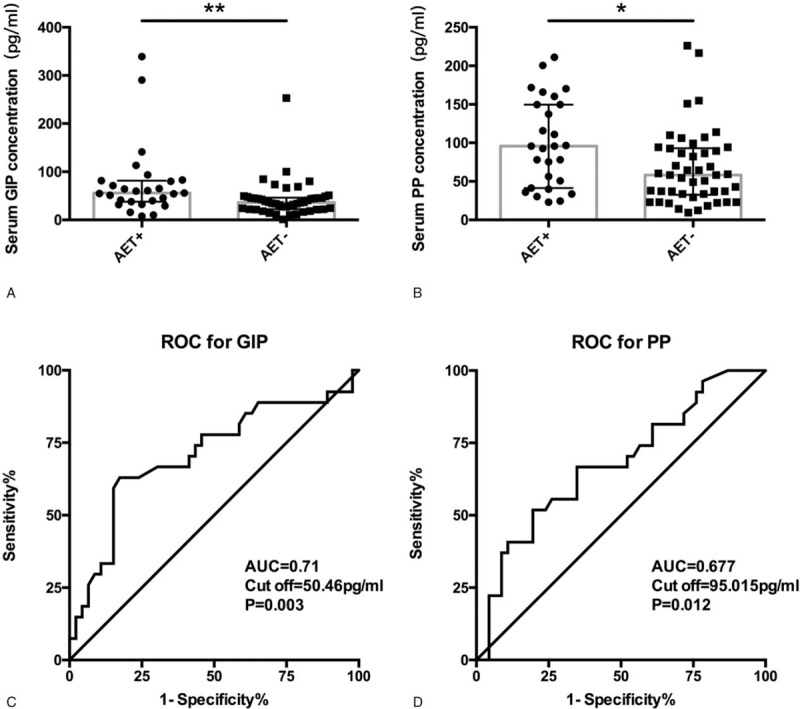
(A and B) AET was independently associated with serum GIP and PP levels (*P* = .003 and .012, respectively). (C) Receiver operating characteristic curve to determine the cut-off value that maximizes the sensitivity and specificity of GIP and PP for predicting AET in GERD. For GIP, the AUC was 0.708, and the optimal cut-off value (50.46 pg/mL) showed a sensitivity of 63% and specificity of 83% (95% CI, 0.58–0.84). (D) For PP, the AUC was 0.68, and the optimal cut-off value (95.02 pg/mL) showed a sensitivity of 52% and specificity of 80% (95% CI, 0.55–0.81). AET = acid exposure time, AUC = area under the ROC curve, CI = confidence interval, GERD = gastroesophageal reflux disease, GIP = gastric inhibitory polypeptide, PP = pancreatic polypeptide.

The median of serum GIP levels were higher in AET+ patients than in AET− patients (55.92 [37.68, 81.58] vs 36.26 [22.13, 46.11] pg/mL, *P* = .003, 2 tailed Mann–Whitnet *U*-test) (Fig. 2A) and the median of serum PP were also higher in AET+ patients than in AET− patients (95.83 [41.32,149.73] vs 58.25 [32.55,92.99] pg/mL, *P* = .012, 2 tailed Mann–Whitnet *U*-test) (Fig. 2B).

The area under the ROC curve (AUC) for the serum GIP level was 0.69, and the optimal cut-off value (50.46 pg/mL) had a sensitivity of 63% and specificity of 83% (95% CI, 0.58–0.84) (Fig. 2C). The AUC for the PP level was 0.68, and the optimal cut-off value (95.02 pg/mL) had a sensitivity of 52% and specificity of 80% (95% CI, 0.55–0.81) (Fig. 2D). The TC level was positively correlated with the GIP level (*r* = 0.28) (*P* = .017). However, waist circumference and fasting blood glucose, TG, HDL-C, and LDL-C levels were not associated with AET.

### 3.3 HRM and impedance and pH parameters

**Figure 3:**
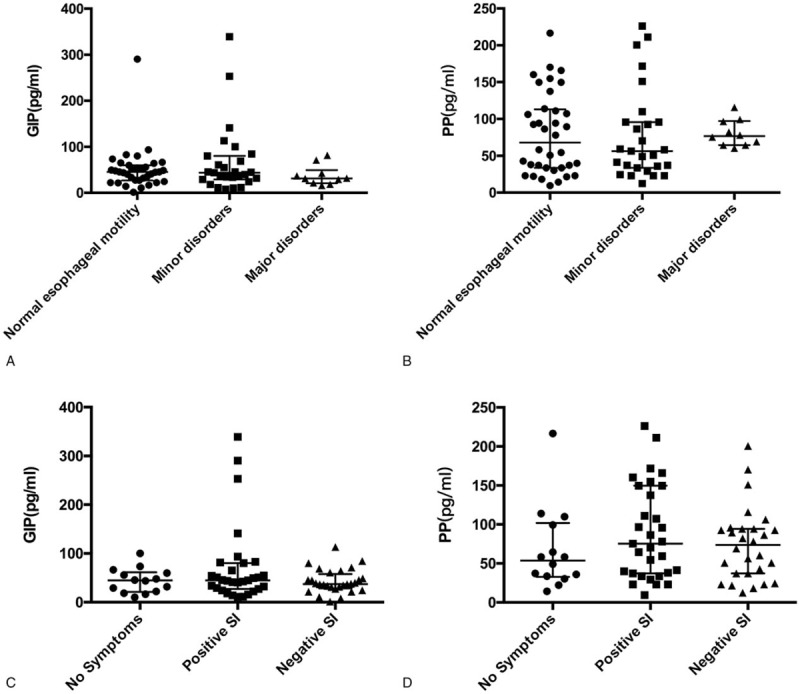
(A and B) Association between Chicago Classification ver. 3.0 and serum GIP and PP levels. (C and D) Serum PP and GIP levels in asymptomatic, positive SI, and negative SI patients during the monitoring period. None of the differences were significant. GIP = gastric inhibitory polypeptide, PP = pancreatic polypeptide, SI = symptom index.

In the AET− group, HRM identified 6 (13.04%) patients with absent contractility, 14 (30.43%) with ineffective esophageal motility (IEM), 2 (4.35%) with fragmental peristalsis, and 24 (52.17%) with normal esophageal motility (Table 2). In the AET+ group, 4 (14.81%) patients had absent contractility, 10 (37.04%) had IEM, 1 (3.70%) had fragmental peristalsis, and 12 (44.44%) had normal esophageal motility. The CFV value was higher in the AET− group (*P* < .05). There was no difference in the DCI, break, DL, IRP4s, UES pressure, or LES pressure between the 2 groups (all *P* > .05). The serum GIP and PP levels did not differ significantly according to CC (Fig. 3A and B).

**Table 2:**
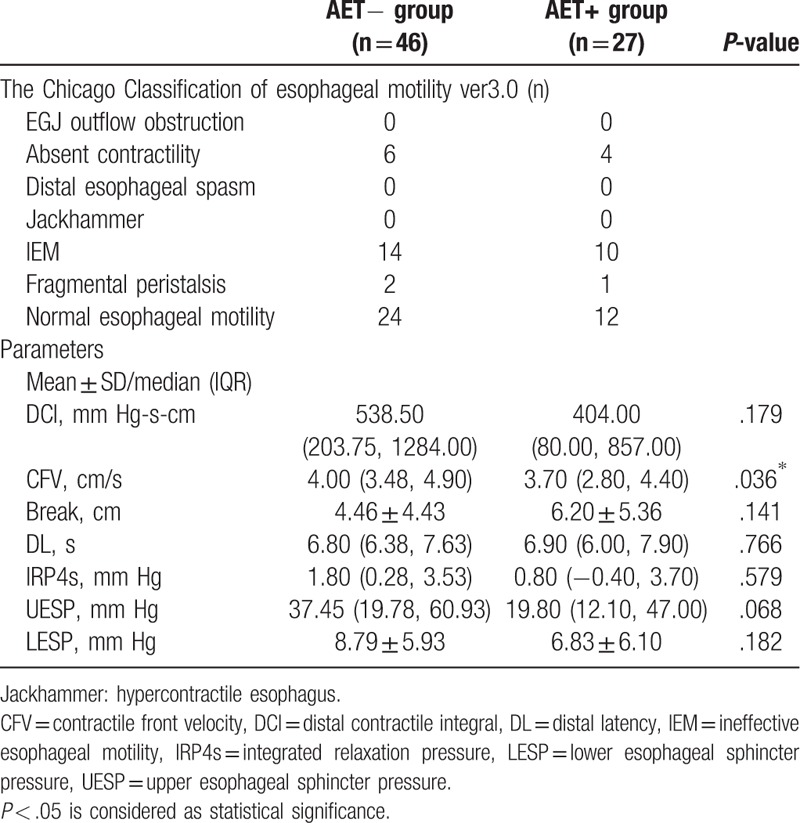
The Chicago Classification and the parameters of esophageal motility and sphincter pressure in HRM.

Of the 73 total subjects, 23 had a DeMeester score >14.72 (pathological acid reflux). One patient each had severe (DeMeester score >100) and moderate pathological acid reflux (50≤ DeMeester score <100). The remaining 21 patients had mild pathological acid reflux (14.72 ≤DeMeester score <50). The median AET was 2.80% (0%–143.40%). The median AET was 3.60% (0%–27.3%) in the upright position and 0.70% (0%–44.4%) in the recumbent position. Circulating levels of GIP (*r* = 0.32) and PP (*r* = 0.24) were positively correlated with AET (*P* < .05), particularly in the upright position (Table 3). However, there was no significant correlation between the reflux substance properties and the GIP or PP level (*P* > .05).

**Table 3:**
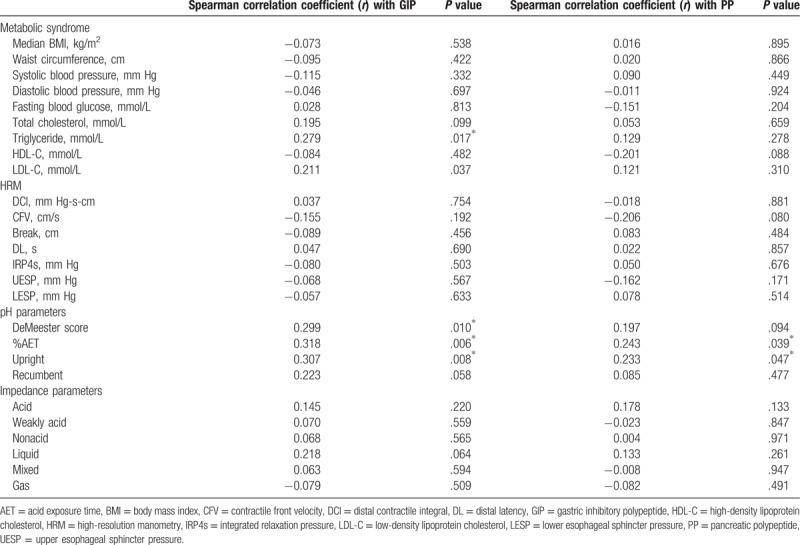
Correlation between GIP and PP levels and metabolic syndrome, HRM, pH-impedance parameters.

Patients with a normal AET and symptoms associated with acid and/or nonacid reflux were regarded as having a hypersensitive esophagus. Patients with a normal AET and no associated symptoms were categorized as having functional heartburn.

A symptom was considered to be associated with reflux if a reflux episode was detected 2 minutes before symptom onset. Fourteen patients did not report discomfort during the monitoring period, 27 patients had a positive SI, and the remaining 32 patients had SI values <50% (Fig. 1). The association with reflux symptoms was not significantly different for serum GIP versus PP level (Fig. 3C and D).

## 4 Discussion and conclusions

The gut–brain axis comprises a network of autonomic neurons that connect the central nervous system, caudal brainstem, and spinal cord to the esophagus, GI tract, liver, and pancreas.^[9]^ As the GI tract mediates ingestion, processing, and absorption of nutrients and energy, the roles of regulatory peptides in the GI tract are important. In previous studies, we tested a series of human metabolic hormones in GERD patients with MILLIPLEX MAP and studied their relationship with esophageal motility and reflux. GIP and PP stood out among these hormones.

GIP is a 42-amino-acid gut-derived peptide secreted by intestinal enteroendocrine K-cells.^[10]^ GIP was discovered in 1969 to 1971 as a result of the recognition that food substances, when introduced into the small intestine, trigger a humoral reflex that inhibits gastric acid secretion. GIP is related to obesity directly in that it increases the volume of adipose tissue and indirectly in that it accelerates fat deposition and expansion of fat depots by increasing insulin secretion from pancreatic β-cells.^[11–13]^

Hormones secreted by pancreatic islet cells also play an important role in GI motility and gastric acid secretion. PP is a 36-amino-acid peptide produced only by islet PP cells.^[14]^ Classically, PP release in response to insulin-induced hypoglycemia is used as an indirect assessment of cholinergic input to islets in humans. However, PP levels respond dramatically to activation of the Vagus nerve by meals or hypoglycemia. Excitation of the Vagus nerve results in increased gastric acid secretion.

Thus, the physiological functions of GIP and PP are related to the secretion of gastric acid. GIP is even a weak inhibitor of secretion of stomach acid and GI motility. Gastric acid reflux to the esophagus is an important cause of GERD, but not unique. RE was assumed to result from the effect of refluxed gastric acid on esophageal squamous epithelial cells.^[15]^ However, the pathogenesis of GERD involves movement of gastric acid to the wrong location, rather than an excessive quantity. Night-time or supine acid reflux is linked to more severe esophagitis.^[16]^ In our study, the serum GIP and PP levels in GERD patients were associated with a significantly increased AET, particularly in the upright position.

An increased GIP level stimulates release of somatostatin and inhibits secretion of gastric acid. Therefore, the acid load in patients with elevated GIP levels should be reduced. Recent research has focused on “gastric acid in the wrong place” and shown that patients with reflux disease do not have more frequent transient LES relaxations (TLESRs) than do controls. Indeed, TLESRs in GERD patients are more likely to be associated with acid reflux.^[17,18]^ Gastric acid is secreted mainly from the upper body and fundus of the stomach, and thus acid may pool in the fundus of a person with GERD and activate sensory nerves, which may enhance TLESRs by facilitating esophageal ingress of acid. Stimulation of esophageal nerves by acid may initiate TLESRs directly, rather than enhancing TLESRs initiated by stomach distention.^[19]^ Therefore, ingress of acid, and not increased acid production, is responsible for activating nerves and increasing GIP secretion via a negative feedback loop. Patients with abnormal esophageal acid exposure may have GIP resistance, similar to insulin resistance. GIP stimulates the release of somatostatin, which inhibits gastric emptying; this reportedly promotes gastric distention and increases TLESR frequency in humans.^[20]^

TLESR is a major mechanism of GERD. However, the relationship between IEM and GERD is controversial. Indeed, IEM, defined as the presence of peristaltic waves at the distal esophagus with an amplitude <30 mm Hg and/or nontransmitted proximal contractions, is frequently observed in GERD patients.^[21]^ However, other studies have reported no association between IEM and GERD.^[22]^ This is in agreement with our finding of no significant correlation among esophageal motility disorders, GIP and PP levels, and AET. The CFV value was higher in the AET– group; however, the role of CFV is not emphasized in the CC ver. 3.0. Our results suggest that GIP and PP do not affect esophageal motility.

We also analyzed the relationships of GIP and PP with reflux-related symptoms. GIP and PP levels were not significantly correlated with nonacidic reflux or liquid, mixed, and gas reflux. Gastric reflux of pH >4 is related to patient discomfort.^[23]^ The pathophysiology of GERD is influenced by the severity and frequency of reflux; yet, severe symptoms may be present in patients with relatively low esophageal acid exposure, and conversely, patients with high acid exposure may have few symptoms.^[24]^

Diabetes mellitus and obesity may also induce or exacerbate GERD.^[25]^ A recent prospective questionnaire study of type 2 diabetes mellitus (T2DM) patients attending an outpatient clinic found GERD symptoms to be 25% more frequent compared within the general population, and were more common in patients with than those without neuropathy.^[26]^ Obesity is a significant independent predictor of symptoms suggestive of gastroparesis, which may lead to GERD, in T2DM patients.^[27]^ Independent of abdominal waist circumference, obesity is associated with increased acid exposure.^[28]^ Chen et al^[29]^ found that increased GIP signaling induces adipose inflammation and impairs insulin sensitivity in mice, which may lead to aggravation of GERD. And GIP mediated through the adipocyte GIP receptor is anabolic in adipose tissue promoting fat deposition. Our data also suggested that the GIP level is positively related to TG levels. However, GIP and PP levels were not significantly correlated with diabetes, BMI, or waist circumference, possibly because diabetes and obesity patients were not targeted in this study. Fasting blood glucose can only represent a temporary blood glucose level, not a long-term level. In the future, the serum GIP and PP levels of patients with T2DM who have been diagnosed with GERD can be included to clarify this issue further. And a larger sample size is needed to perform more robust analyses.

The present study had several limitations. First, the sample size was relatively small, and the number of patients in each GERD category, particularly those with Barret's esophagus, was limited. A lack of sufficient statistical power to demonstrate differences among the groups could have led to type II errors. Second, due to the high cost and poor tolerance of HRM and 24 hours MII-pH monitoring, we were unable to compare patients with diabetes, obese patients, and patients with GERD. Third, the mechanism underlying the changes in GIP and PP levels in AET+ patients need to be evaluated. Finally, as this study was cross-sectional in design, the causal link between the function of the respective hormones and the spectrum of GERD could not be established. Further longitudinal or interventional studies are thus warranted.

In conclusion, serum GIP and PP levels are related to prolonged acid exposure, particularly in the upright position. Due to the limitations and cost of 24 hours MII-pH monitoring, serum GIP and PP may be used as markers predicting prolongation of AET. Further prospective studies involving GERD patients with diabetes and obesity are needed. The mechanism underlying the roles of GIP and PP in GERD will be evaluated using animal models and in in vitro experiments.

## Author contributions

Jing Chen designed the study, analyzed and drafted the manuscript; Baona Guo and Zihao Guo collected data and interpreted data; Li Li and Jiali Jiang collected data, interpreted data. Jixiang Wu critically revised the manuscript for important intellectual content; Yutao Zhan and Chuan Zhang designed the study and interpreted data and critically revised the article for important intellectual content.

**Conceptualization:** Chuan Zhang.

**Formal analysis:** Jiali Jiang.

**Funding acquisition:** Jixiang Wu.

**Investigation:** Baona Guo.

**Methodology:** Jing Chen.

**Project administration:** Yutao Zhan.

**Resources:** Li Li.

**Software:** Jing Chen.

**Writing – original draft:** Jing Chen.

**Writing – review and editing:** Zihao Guo, Yutao Zhan.
